# Good Cause for Suicide

**Published:** 1893-05

**Authors:** 


### Good Cause for Suicide.

William Harman, a resident of Titusville, Pa., committed suicide a few days ago from a melancholy conviction that he was his own grandfather. Here is a singular letter that he left. "I married a widow who had a grown-up daughter. My father visited our house very often, fell in love with my step-daughter and married her. So my father became my son-in-law and my step-daughter my mother, because she was my father's wife. Some time afterwards my wife had a son; he was my father's brother-in-law and my uncle, for he was the brother of my step-mother. My father's wife, *i. e.*, my step-daughter, had also a son; he was of course, my brother, and in the meantime my grandchild, for he was the son of my daughter. My wife was my grandmother, because she was my mother's mother. I was my wife's husband and grandchild at the same time. And as the husband of a person's grandmother is his grandfather, I was my own grandfather."—*Montreal Medical Journal.*

